# Metabolic signatures of osteoarthritis in urine using liquid chromatography‐high resolution tandem mass spectrometry

**DOI:** 10.1007/s11306-021-01778-3

**Published:** 2021-03-03

**Authors:** Salah Abdelrazig, Catharine A. Ortori, Michael Doherty, Ana M. Valdes, Victoria Chapman, David A. Barrett

**Affiliations:** 1 Centre for Analytical Bioscience, Advanced Materials and Healthcare Technologies Division, School of Pharmacy, University of Nottingham, Nottingham, NG7 2RD UK; 2 Pain Centre Versus Arthritis, Queen’s, Medical Centre, Medical School, University of Nottingham, Nottingham, NG7 2RD UK; 3 School of Medicine, University of Nottingham, Nottingham, NG7 2RD UK; 4 NIHR Nottingham Biomedical Research Centre, University of Nottingham, Nottingham, NG7 2RD UK; 5 Centre for Sport, Exercise and Osteoarthritis Research Versus Arthritis, University of Nottingham, Nottingham, NG7 2RD UK; 6 School of Life Sciences, University of Nottingham, Nottingham, NG7 2RD UK

**Keywords:** Osteoarthritis, Untargeted metabolomics, LC-HRMS, Biomarkers, HILIC

## Abstract

**Introduction:**

Osteoarthritis (OA) is a common cause of disability in older people, but its aetiology is not yet fully understood. Biomarkers of OA from metabolomics studies have shown potential use in understanding the progression and pathophysiology of OA.

**Objectives:**

To investigate possible surrogate biomarkers of knee OA in urine using metabolomics to contribute towards a better understanding of OA progression and possible targeted treatment.

**Method:**

Liquid chromatography-high resolution mass spectrometry (LC-HRMS) was applied in a case–control approach to explore the possible metabolic differences between the urinary profiles of symptomatic knee OA patients (n = 74) (subclassified into inflammatory OA, n = 22 and non-inflammatory OA, n = 52) and non-OA controls (n = 68). Univariate, multivariate and pathway analyses were performed with a rigorous validation including cross-validation, permutation test, prediction and receiver operating characteristic curve to identify significantly altered metabolites and pathways in OA.

**Results:**

OA datasets generated 7405 variables and multivariate analysis showed clear separation of inflammatory OA, but not non-inflammatory OA, from non-OA controls. Adequate cross-validation (R^2^Y = 0.874, Q^2^ = 0.465) was obtained. The prediction model and the ROC curve showed satisfactory results with a sensitivity of 88%, specificity of 71% and accuracy of 77%. 26 metabolites were identified as potential biomarkers of inflammatory OA using HMDB, authentic standards and/or MS/MS database.

**Conclusion:**

Urinary metabolic profiles were altered in inflammatory knee OA subjects compared to those with non-inflammatory OA and non-OA controls. These altered profiles associated with perturbed activity of the TCA cycle, pyruvate and amino acid metabolism linked to inflammation, oxidative stress and collagen destruction. Of note, 2-keto-glutaramic acid level was > eightfold higher in the inflammatory OA patients compared to non-OA control, signalling a possible perturbation in glutamine metabolism related to OA progression.

**Supplementary Information:**

The online version contains supplementary material available at 10.1007/s11306-021-01778-3.

## Introduction

Osteoarthritis (OA) is the most common form of arthritis (Chen et al. 2017). The World Health Organisation estimated that 9.6% of men and 18% of women above 60 years of age have symptomatic OA worldwide (WHO, 2020). The exact aetiology of OA is not yet fully understood. However, it is recognised to be a common complex disorder with multiple genetic, constitutional and environmental risk factors including increasing age, female gender, obesity and joint usage and trauma (De Ceuninck et al. 2011; Li et al. 2010). Current OA research is directed towards prevention, early diagnosis, and understanding of the aetiology and progression of OA, with the hope that better understanding will lead to better treatment options (Hunter et al. 2019).

To date, no disease-modifying agent has been approved to effectively treat OA and therefore, treatment strategies aim to improve the quality of life through management of symptoms including pain control and improvement of reduced function (Mora et al. 2018). Currently, OA is diagnosed predominantly according to characteristic joint symptoms and abnormal signs, together, if required, with imaging evidence of OA structural changes using radiography (the usual, most widely available modality), ultrasonography (increasingly available but not as comprehensive as MRI in its tissue assessment) or MRI (very sensitive and comprehensive, but costly and least available) (NICE guidelines) (Loeuille, 2012; Menashe et al. 2012). These techniques lack the ability to provide pathophysiological information at early stages of OA development. Therefore, the identification of new OA biomarkers using alternative technologies might aid in the search for the development of new diagnostic tests and the identification of new drug targets.

Untargeted metabolomics aims to provide an unbiased overview of the metabolic patterns in a biological system and may provide a direct way to identify surrogate biomarkers and study the underlying perturbations of metabolic pathways in the clinical progression of OA. Urine is readily available for non-invasive sampling and provides an end metabolite pool of the body. Hence, it has the potential to increase our understanding of the metabolic variation associated with OA development and progression. There are relatively few studies related to the use of urine metabolomics in OA (Lamers et al. 2003, 2005; Li et al. 2010; Nepple et al. 2015). These studies showed that metabolites related to tricarboxylic acid (TCA), histamine, purine and energy metabolism are associated with OA and could be logically linked to the disturbed biochemical pathways of the condition. Therefore, further research is needed to identify, confirm, validate and characterise OA urinary biomarkers. The use of liquid chromatography-high resolution mass spectrometry (LC-HRMS) employing hydrophilic interaction chromatography (HILIC) is preferred for the analysis of polar and semi-polar metabolites typically found in urine (Buszewski & Noga, 2012; WHO, 2020).

In this study, we applied untargeted metabolomics using HILIC LC-HRMS analysis to urine samples collected from OA patients and non-OA controls. We investigated alterations in urinary metabolic end-products related to perturbations in the metabolic pathways in OA and attempted to relate these changes to biochemical pathways relevant to OA disease and contribute to the understanding of OA progression.

## Materials and methods

### Reagents and chemicals

Reagents, chemicals and 171 authentic standards used for the LC-HRMS optimisation, validation and/or metabolite identification were either HPLC or MS grade; their description and details are summarized in Tables S1 and S2.

### Ethics approval, sample collection and storage

The study was approved by the Nottingham University Hospital Research Ethics Committee 1 (NRES reference 14/EM/0013), and fully informed written consent was obtained from participants prior to study entry. Community-derived 142 participants aged over 30 years comprised: (1) people with symptomatic knee OA (n = 74), having predominantly usage-related pain plus definite radiographic joint space narrowing and osteophyte in at least one knee compartment (using a single standardised semi-flexed weight-bearing tibio-femoral view and a Rosen template to control knee flexion and foot external rotation, and skyline 30-degree flexion patella-femoral views); and (2) control non-OA participants (n = 68) with no knee pain and no clinical or radiographic signs of knee OA. Participants with knee OA were classified into two phenotypes based on clinical assessment: those with inactivity stiffness plus joint line tenderness and either grade 2 effusion (positive “balloon sign” for fluctuance) or morning stiffness were classified as “inflammatory OA” (n = 22), whereas those not fulfilling this definition were classified as “non-inflammatory OA (n = 52). Exclusion criteria for both groups included clinically significant disease affecting the endocrine, hepatic, cardiac, respiratory, or renal systems. However, some subjects with comorbidities of heart attack history (12%), stroke (4.9%), epilepsy (1.4%), hypertension history (33%), asthma (7.7%), psoriasis (2.1%), irritable bowel syndrome (4.9%), thyroid problems history (9.2%), diabetes (8.5%), kidney problems history (5.6%), liver problems history (2.1%), gout (7.7%), osteoporosis (1.4%), depression (15.5%), cancer history (15.5%), fibromyalgia (2.8%) and chronic fatigue syndrome (0.7%) were included in the study. Fasting (i.e. no breakfast) morning urine samples were collected from all the participants without the use of preservatives, aliquoted in 2.0 mL triplicates and stored immediately at − 80 ˚C.

### Preparation of urine samples for LC-HRMS

The urine samples (60 µL) were centrifuged at 10,000×*g* for 10 min, 50 µL of the supernatant was diluted with 100 µL water and analysed using LC-HRMS. Blanks were prepared in the same way without including urine sample. For metabolomics analysis, pooled QC samples were prepared by mixing 20 µL from each urine sample in the study.

### LC-HRMS and LC-HRMS/MS analyses

Chromatography was performed on Accela UHPLC system/Dionex U3000 (Thermo Fisher Scientific, Hemel Hempstead, UK) on a BEH HILIC column (2.1 × 100 mm, 1.7 µm particle size; Waters, Milford, USA) coupled to an orbital trap mass spectrometer (Exactive/Q-Exactive, Thermo Fisher Scientific, Hemel Hempstead, UK). The column was maintained at 400 µL/min, 40 °C. Mobile phases used were: (A) 50:50 and (B) 95:5 acetonitrile:ammonium acetate (10 mM in water). Urine samples (5 µL, 4 °C) were injected in a ramp gradient from 1% (A) to 100% (A) in 12 min then the composition was returned to its initial conditions and maintained for the second run (15 min). The MS parameters were optimised for urine analysis using the standard mixture (Table S1) of a selected set of 35 urinary metabolites (Bouatra et al. 2013). LC-HRMS was performed using Exactive-MS in simultaneous ESI + and ESI − modes. The operational parameters of MS were spray voltage 3.2 kV (ESI +), 2.4 kV (ESI −), capillary voltage 25 V (ESI +), − 27 V (ESI −). Sheath, auxiliary and sweep gas flow rate were 20, 5 and 5 (arbitrary unit), respectively, for both modes. Capillary and heater temperature were maintained at 350 and 120 °C, respectively. Data were acquired in full scan mode with resolution 50,000, AGC 1e^6^ from *m/z* 60–1000 with 4 Hz scan rate. Metabolite identification was performed on the pooled QC sample (n = 3) and co-analysed with 171 authentic standards using Q-Exactive MS with Top 5 ddMS/MS scans at a resolution of 17,500 and a stepped normalised collision energy (NEC) of 20, 30 and 40.

### Urinary metabolomics analysis of participants with OA and non-OA controls

Urine samples in the study were randomised and analysed in a single batch with LC-HRMS. The mixture of authentic standards (Table S1) were 10 times diluted and co-analysed with the samples as a reference test mix to check the performance and the stability of the instrument. The pooled QC was also injected at the beginning to condition the column and every 5–10 samples to monitor the stability, robustness, repeatability and performance of the LC-HRMS.

### Data analysis and metabolite identification

#### LC-HRMS validation

The performance of the analysis was validated by principal component analysis (PCA) and by monitoring the variability in the response of a representative set of 54 metabolites in the set of pooled QC samples. In addition, the quality of the acquired datasets was assessed by determining the relative standard deviation (RSD) of the peak areas of all peaks present in at least 80% of the pooled QC (Want et al. 2010).

#### Multivariate analysis

The raw datasets were pre-processed with Progenesis QI (Nonlinear Dynamics, Waters, Milford, USA), normalised using MS total useful signal (MSTUS) (Warrack et al. 2009) and ArcSinh transformed (Jones, 2008) to restore normality to the datasets. Any detected ions related to analgesics received by the OA participants were identified as detailed in the metabolite identification section and excluded manually to minimise the potential confounding effect of medication in the classification of the OA samples. Simca P + 13/ + 14 (Umetrics, Umeå, Sweden) was used for multivariate analysis in which PCA and orthogonal partial least squares-discriminant analysis (OPLS-DA) were generated to investigate any possible trends and metabolic changes between OA participants and non-OA controls. Shared and unique structures (SUS) plot was used to balance the sample size in each class as stated elsewhere (Kirwan et al. 2012). The robustness of the OPLS-DA models was evaluated by cross-validation (R^2^Y: fitness of model, Q^2^: predictive ability), permutation test, prediction (50:50 training:test sets) using Simca P random selection function (Eriksson et al. 2006a) and area under the receiver operating characteristic (ROC) curve (AUC). Variable Importance for the Projection (VIP) and *p*(corr) of the OPLS-DA and *p*-values from Student’s *t*-Test, adjusted using Benjamini and Hochberg false discovery rate, were used to select the ions responsible for the class separation between the OA participants and the non-OA controls.

#### Metabolite identification

The QC samples analysed with top five MS/MS transitions were processed by Compound Discoverer 3.1 SP1 (Thermo Fisher Scientific, Hemel Hempstead, UK) for metabolite identification. The significantly altered urinary metabolites in the study were identified by matching their *m/z*, retention times (RTs) and MS/MS spectra with the metabolites in Human Metabolome Database (HMDB) (Wishart et al. 2018) and/or the MS/MS of standards*, mz*Cloud and *mz*Vault database (Thermo Fisher Scientific, Hemel Hempstead, UK). The identified metabolites were then classified based on the confidence in identification recommended by Chemical Analysis Working Group, Metabolomics Standards Initiative (MSI) (Sumner et al. 2007). In MSI scale, metabolites were either classified as level 1: Identified compounds with reference standards (*m/z*, RT and MS/MS), level 2: putatively annotated compounds based on the use of spectral library and no reference standards (*m/z* and/or MS/MS), level 3: putatively characterised compound classes and level 4: unknowns.

## Results

### Assessment of the demographic data for metabolomics analysis

The median age of participants in the study was 68 years old (range: OA participants 50–91; non-OA controls 52–88), indicating adequate age matching for metabolomics analysis (Table 1). There was no significant difference (*p*-value = 0.05) in body mass index (BMI) between the OA participants and the non-OA controls, indicating that they were well matched for the study. However, a significant difference in BMI (*p*-value = 0.02) was observed between the inflammatory OA participants and the non-OA controls.

**Table 1:** Demographic data comparing the OA participants to the non-OA controls

Description	OA participants	Non-OA controls
1. Number of participants		
a. All	74	68
Male	26	30
Female	48	38
b. Inflammatory OA participants	22	–
Male	5	–
Female	17	–
c. Non-inflammatory OA participants	52	–
Male	21	–
Female	31	–
2. Age		
a. All		
Median	68	68
Range	50–91	52–88
b. Inflammatory OA participants		
Median	69	–
Range	54–86	–
c. Non-inflammatory OA participants		
Median	68	–
Range	50–91	–
3. BMI		
a. All		
Mean (*p*-value* = 0.05)	30.23	28.34
Median	29.27	27.62
Range	20.40–51.04	20.28–45.52
b. Inflammatory OA participants vs non-OA controls		
Mean (*p*-value = 0.02)	31.55	28.34
Median	29.41	27.62
Range	24.77–46.85	20.28–45.52
c. Non-inflammatory OA participants vs non-OA controls		
Mean (*p*-value = 0.19)	29.67	28.34
Median	28.99	27.62
Range	20.40–51.04	20.28–45.52

*p-values were computed using a two tail Student’s *t*-Test at 95% confidence limits assuming equal variance between groups. *BMI* body mass index, *OA* osteoarthritis, *vs* versus

### The performance of the LC-HRMS for untargeted metabolomics

**Fig. 1:**
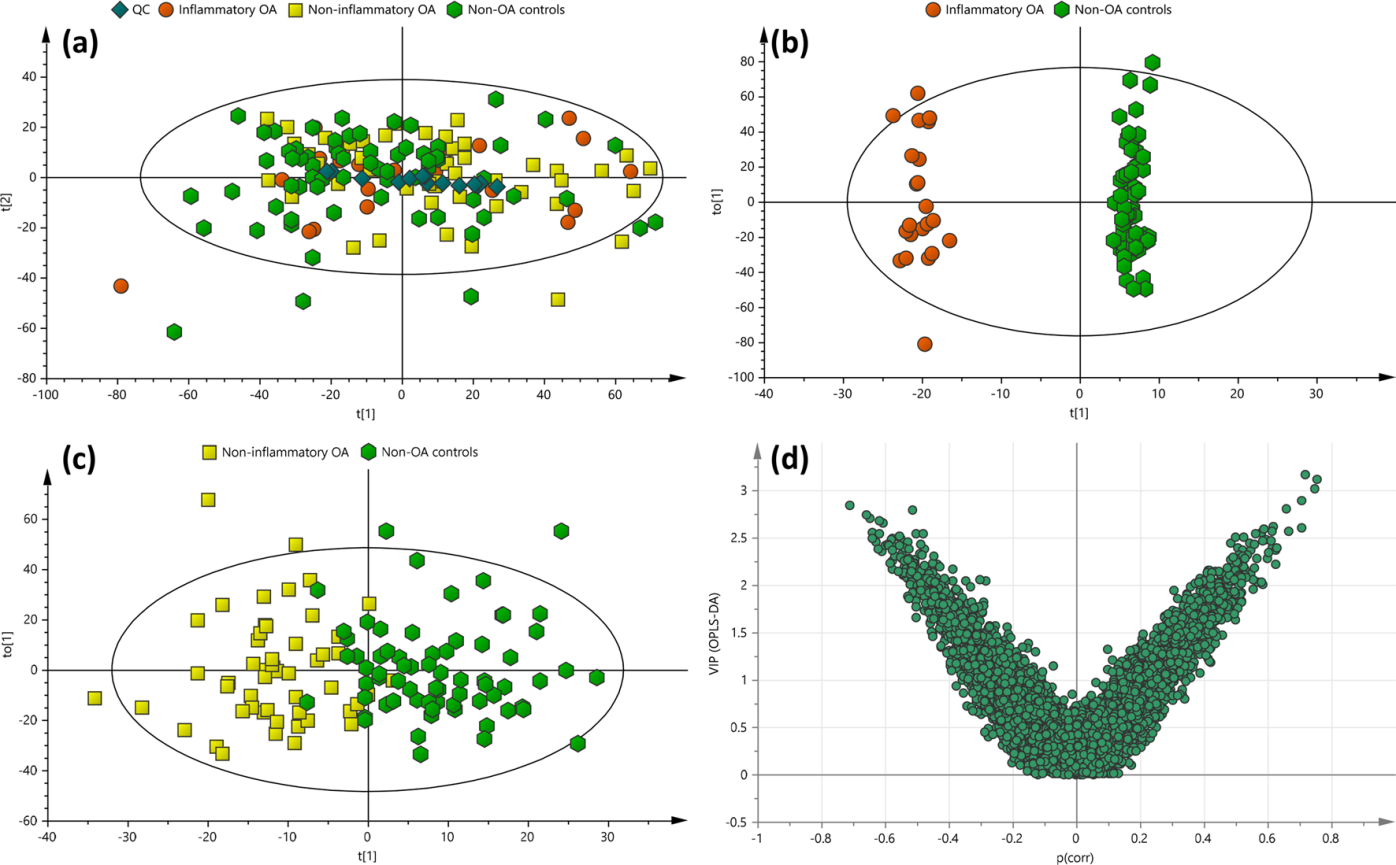
PCA and OPLS-DA score plots obtained from all OA participants and non-OA controls. **a** PCA of non-OA controls (n = 68), inflammatory OA (n = 22), non-inflammatory OA (n = 52) participants and pooled QCs (n = 15), whereas **b** OPLS-DA of inflammatory OA participants and non-OA controls and **c** OPLS-DA of non-inflammatory OA participants and non-OA controls analysed by LC-HRMS. **d** The Significantly altered metabolites were selected using VIP vs *p*(corr) of OPLS-DA of inflammatory OA participants and non-OA controls

The quality of the LC-HRMS datasets in the study was assessed using the QC samples in which the RSDs of the selected 54 ions were within 1% for RTs and 18% (range 7–18%) for peak areas in the QC (Table S3). The RSDs across the mean peak areas of at least 80% of all peaks present in the QC were less than 30% for 71% of these peaks, which were lower than the recommended threshold for metabolomics analysis (Begou et al. 2018). PCA was also used to assess the quality of the acquired datasets; the QC samples were adequately clustered towards the centre of the PCA score plot (Fig. 1a). These results demonstrate satisfactory stability and validate the LC-HRMS for urine metabolomics.

Visual examination of the LC-HRMS base peak chromatograms (BPCs) of urine samples showed differences between the different classes in the study. For instance, in Figure S1, the marked regions (A) and (B) show an increased level of the metabolite, *m/z* 232.0274 (unknown) and *m/z* 152.0706 (phenylglycine) in inflammatory OA participants compared to non-inflammatory OA participants and non-OA controls. The metabolite ion, *m/z* 181.0286 (ESI +) and, *m/z* 286.2642 (ESI −) showed higher peak areas in the inflammatory OA compared to the non-inflammatory OA participants and non-OA controls. Creatinine, *m/z* 114.0667 (ESI +) was found to be the most abundant ion in the urine sample of non-OA controls. Taking all molecular features into account, most of the detected ions were concentrated in the lower mass range (*m/z* 60–300) in the positive mode, while relatively higher mass ions were detected in the negative modes.

### Data analysis

**Fig. 2:**
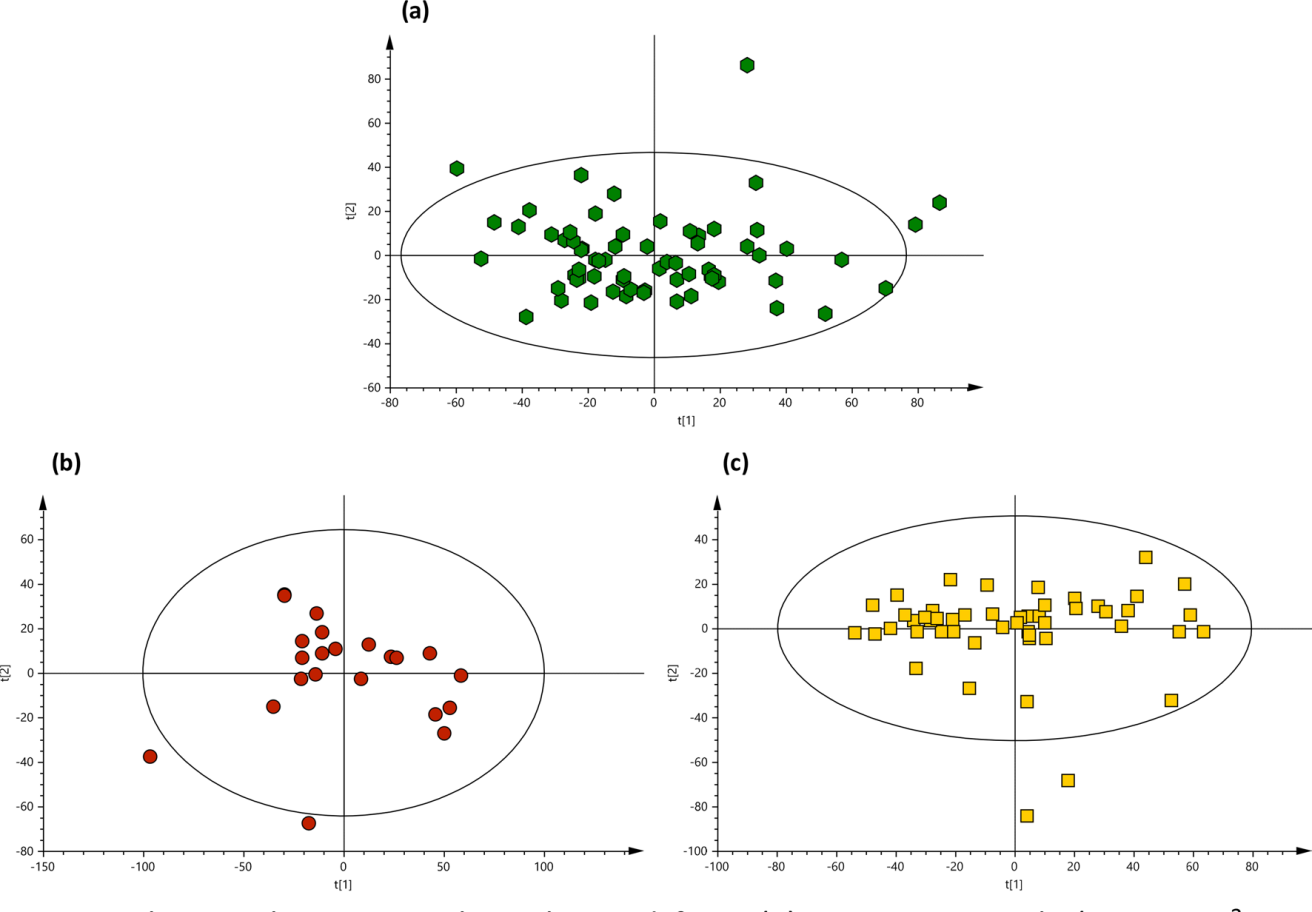
PCA-class analysis score plots obtained from **a** non-OA controls (n = 68, R^2^X = 0.39, Q^2^ = − 0.001), **b** inflammatory OA (n = 22, R^2^X = 0.35, Q^2^ = − 0.014) and **c** non-inflammatory OA (n = 52, R^2^X = 0.42, Q^2^ = − 0.006) participants’ urine samples analysed by LC-HRMS

**Fig. 3:**
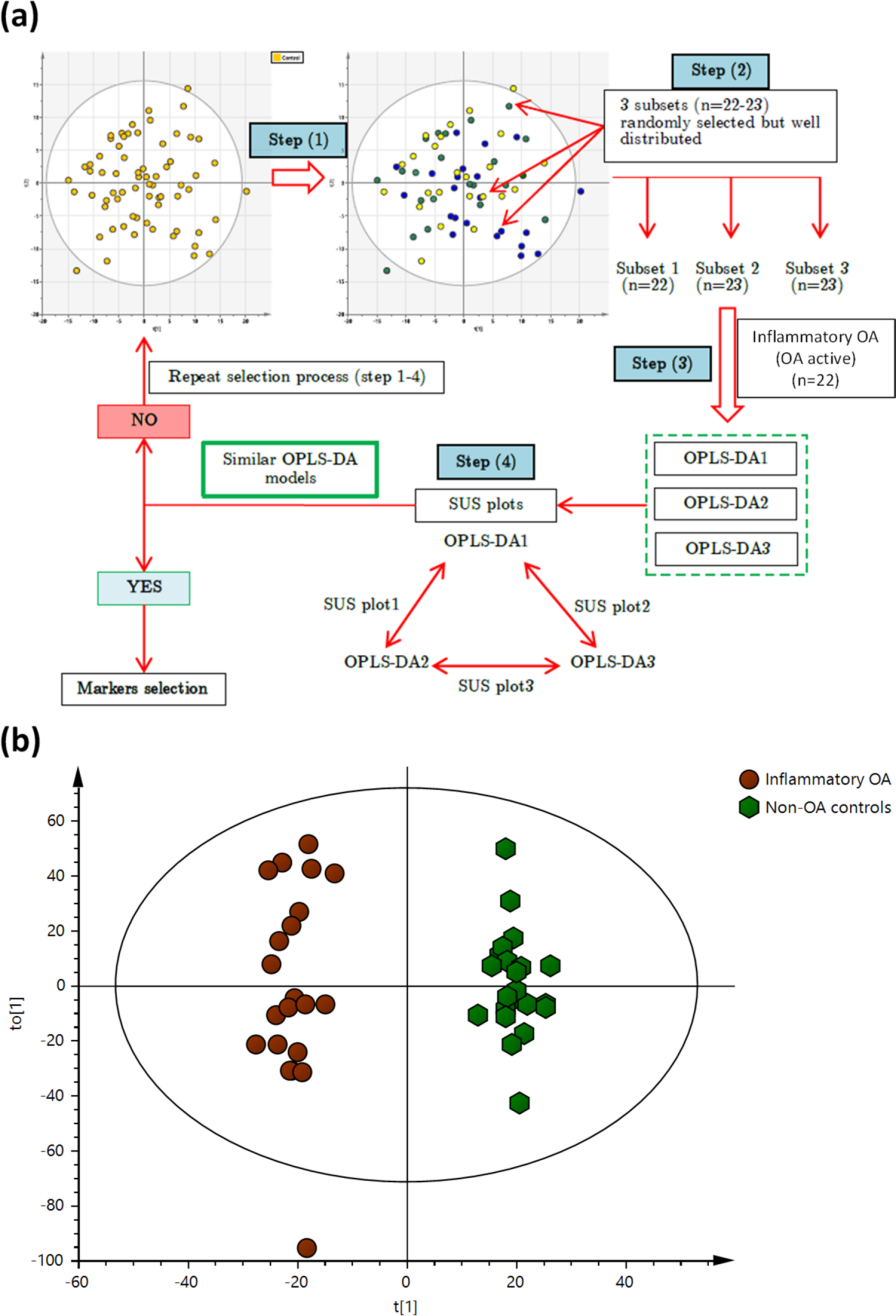
Workflow for balancing class size of non-OA control with inflammatory OA for biomarker analysis. **a** Non-OA control (n = 68) were sub-divided into 3 subsets using multivariate design based on PCA single class analysis. 3 OPLS-DA models were generated from each dataset against inflammatory OA patients’ dataset. SUS plot was used to monitor the similarity of the generated OPLS-DA models. SUS plots were generated for 2 models at a time. This procedure was repeated until the selected subsets of the healthy controls generated adequately similar OPLS-DA models with inflammatory OA patients. **b** OPLS-DA score plot obtained from inflammatory OA patients (OA active) and the balanced non-OA controls urine samples

The metabolomics datasets of OA patients and non-OA controls generated 7405 features and were submitted for multivariate and univariate analyses. PCA-class analysis was first performed to evaluate the similarity of the samples within each class. Adequate similarity with no significant differences were observed between the samples in each class indicated by the poor PCA-class Q^2^ values in all classes (Q2 < 0.01, Fig. 2). This demonstrates that the underlying comorbidities in some subjects had no significant clustering or separation in the metabolic profile within each class. No separation or clustering was observed in the PCA between the different classes in the study (Fig. 1a), hence, subsequent OPLS-DA were constructed. A complete separation was observed in the OPLS-DA between the inflammatory OA participants and the non-OA controls (Fig. 1b) indicating significant difference with good R^2^Y (0.874) and Q^2^ (0.465). Univariate analysis was performed in parallel to multivariate analysis in which the adjusted *p*-values from Student’s *t*-Test were computed across all ions in OA participants compared to non-OA controls to identify significantly altered metabolites related to OA, if any. 26 metabolite features were found significantly different in inflammatory OA participants compared to non-OA controls (Table 2). SUS plots and balanced OPLS-DA were used to demonstrate that the small change in BMI in inflammatory OA compared to non-OA controls was not related to the significant metabolic features found between the two classes. An equal set of samples from inflammatory OA (n = 22) vs no-OA controls (n = 22) were randomly selected to minimise bias using SUS plot and subsequently a balanced OPLS-DA was generated (Fig. 3). No significant difference in BMI (i.e. *p*-value = 0.05) was observed between the 2 classes and the reported metabolites were found still significant, hence not related to BMI. Insufficient separation was observed in the OPLS-DA of non-inflammatory OA participants and non-OA controls with very poor Q^2^ of − 0.221, indicating no significant difference between the two classes (Fig. 1c).

**Table 2:** The identified urinary biomarkers of the inflammatory OA participants

Metabolite	Formula	RT (min)	Fold Change^a^	Adjusted *p*-value	VIP score	Identification^b^	
						Method	Level
2,3-Diaminopropionic acid	C_3_H_8_N_2_O_2_	1.50	1.9	0.0267	2.8	Accurate mass	2
2-Keto-glutaramic acid	C_5_H_7_NO_4_	4.06	8.24	0.0045	1.9	Accurate mass	2
2-Hydroxyhippuric acid	C_9_H_9_NO_4_	4.10	− 2.3	0.0308	1.6	Accurate mass, MS/MS	2
3-Methoxyphenylacetic acid	C_9_H_10_O_3_	0.60	− 3.4	0.0484	2.6	Accurate mass, MS/MS	2
3-Methylcrotonylglycine	C_7_H_11_NO_3_	5.16	− 2.1	0.0356	2.4	Accurate mass	2
3-Nitrotyrosine	C_9_H_10_N_2_O_5_	1.68	1.4	0.0421	2.4	Accurate mass	2
3-Oxoalanine	C_3_H_5_NO_3_	2.27	− 1.5	0.0462	2.4	Accurate mass	2
4-Hydroxybutyric acid	C_4_H_8_O_3_	1.36	− 1.7	0.0073	3.0	Accurate mass, RT, MS/MS	1
Acetylphosphate	C_2_H_5_O_5_P	7.89	1.3	0.0254	1.3	Accurate mass	2
Aminoadipic acid	C_6_H_11_NO_4_	1.36	− 1.3	0.0186	2.8	Accurate mass, MS/MS	2
Creatinine	C_4_H_7_N_3_O	3.16	− 1.2	0.0004	2.4	Accurate mass, RT, MS/MS	1
Cytosine	C_4_H_5_N_3O_	2.47	− 1.4	0.0484	2.5	Accurate mass, RT, MS/MS	1
Fumarate	C_4_H_4_O_4_	13.65	1.4	0.0362	1.5	Accurate mass, RT, MS/MS	1
Homocysteine sulphinic acid	C_4_H_9_NO_4_S	1.47	− 3.5	0.0462	2.1	Accurate mass	2
Hydroxykynurenine	C_10_H_12_N_2_O_4_	1.45	− 2.6	0.0356	2.2	Accurate mass	2
Hypoxanthine	C5H_4_N_4_O	1.99	− 1.7	0.0486	2.1	Accurate mass, RT, MS/MS	1
l-Homoserine	C_4_H_9_NO_3_	1.49	− 1.3	0.0086	2.9	Accurate mass, RT, MS/MS	1
N-Acetyl-l-glutamate 5-semialdehyde	C_7_H_11_NO_4_	1.52	− 1.6	0.0032	3.1	Accurate mass	2
N-Phenylacetyl-l-glutamine	C_13_H_16_N_2_O_4_	1.68	− 1.4	0.0421	2.3	Accurate mass, MS/MS	2
Phosphoric acid	H_3_O_4_P	1.49	1.8	0.0421	2.5	Accurate mass, MS/MS	2
Pipecolic acid	C_6_H_11_NO_2_	2.88	-2.7	0.0395	1.5	Accurate mass, MS/MS	2
Prolyl-Glutamate	C_10_H_15_N_2_O_5_	1.57	2.3	0.0484	2.4	Accurate mass	2
Pyruvic acid	C_3_H_4_O_3_	7.75	− 1.1	0.0056	1.4	Accurate mass, RT, MS/MS,	1
S-Lactoylglutathione	C_13_H_21_N_3_O_8_S	4.75	1.5	0.0405	2.5	Accurate mass	2
Suberic acid	C_8_H_14_O_4_	5.31	1.1	0.0214	1.0	Accurate mass, MS/MS	2
Tryptophan	C_11_H_12_N_2_O_2_	1.68	− 1.4	0.0301	2.4	Accurate mass, RT, MS/MS,	1

^a^Fold change: ( +): increased and (−) decreased level of the metabolite in inflammatory OA compared to non-OA controls

^b^Levels of confidence in identification (1–4) was based on MSI method as previously detailed

The OPLS-DA of inflammatory OA participants and non-OA controls was further validated using a permutation test, prediction and AUC, the validation results are listed in Table S4. In the permutation test, the regression line of the Y-permuted Q^2^ was intercepted at − 0.130, indicating a reliable predictive power of the model (Eriksson et al. 2006b). Prediction showed a satisfactory model with a sensitivity of 88%, specificity of 71% and accuracy of 77%. The sensitivity and specificity of this model were further assessed by computing the AUC which was 0.76 indicating a good clinical utility for biomarker discovery (Xia et al. 2013). These results validate the model.

### Selection and identification of potential biomarkers of OA in urine

Significantly altered metabolites between the inflammatory OA participants and non-OA controls were selected using VIP score > 1.0, │*p*(corr)│ > 0.4 and q-value < 0.05 (Fig. 1d). 26 metabolites were identified as significantly altered metabolites in inflammatory OA participants compared to non-OA controls (Table 2). The MS/MS spectral matching of the metabolites are illustrated in Figures S2 to S17. Some of the significantly changed metabolites remained putatively identified, however, they remain listed due to their potential importance for interpretation of the study data.

### Pathway analysis

**Fig. 4:**
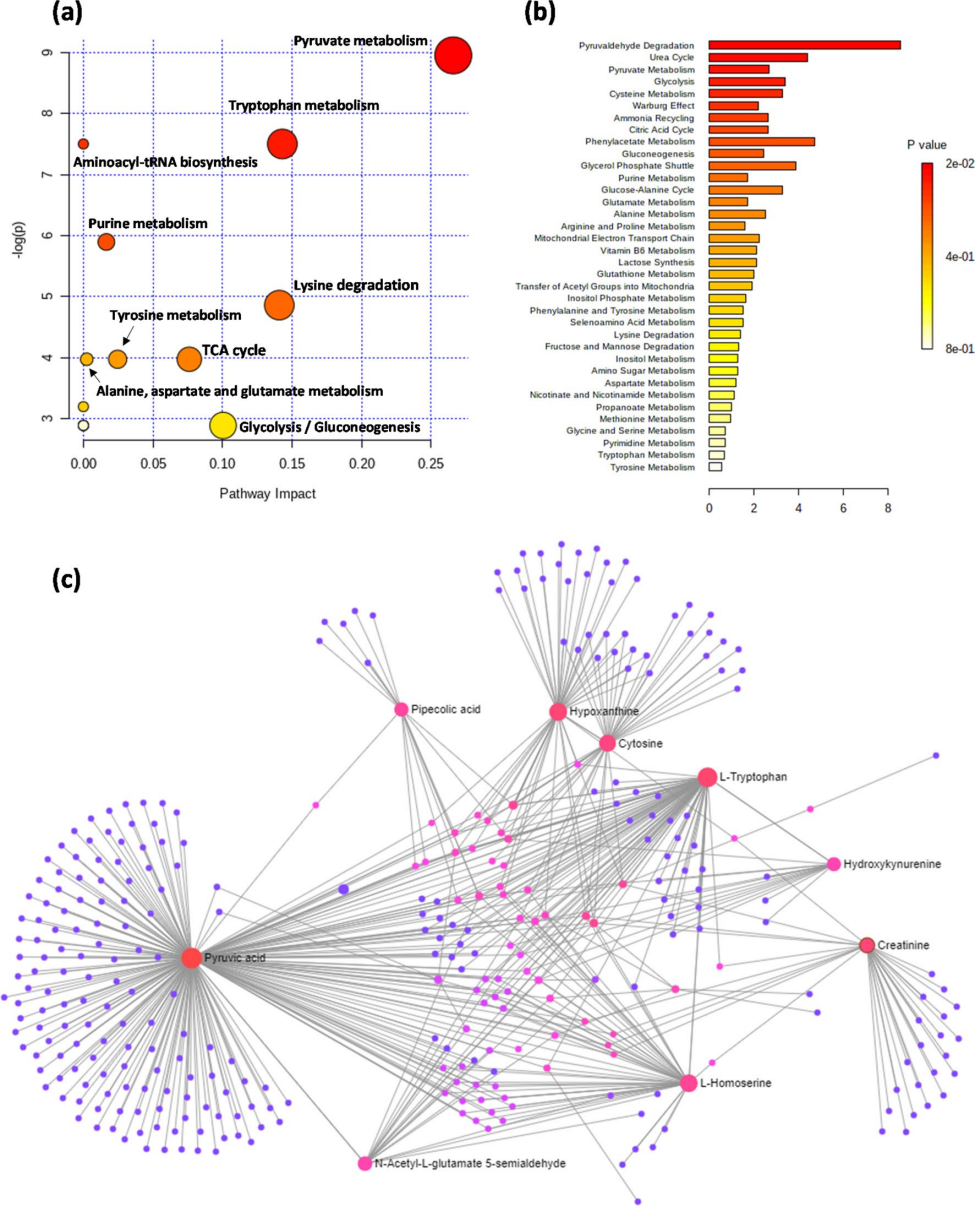
Pathway analysis of the significantly altered metabolites in inflammatory OA participants compared to non-OA controls. **a** Pathway analysis, **b** pathway enrichment analysis and **c** pathway network map highlighting significantly changed pathways and interactions between the significantly altered metabolites in inflammatory OA participants compared to non-OA controls

The normalised abundances of the significantly altered metabolites in inflammatory OA participants and non-OA controls were processed for pathway enrichment, analysis and network mapping using MetaboAnalyst 4.0 (Chong et al. 2019). Different metabolic pathways (Table S5) including pyruvate, purine and lysine metabolism were found significantly altered in inflammatory OA participants compared non-OA controls as illustrated in Fig. 4.

## Discussion

Significant differences in the urinary profile of inflammatory OA participants were found compared to the non-OA controls in which 26 metabolites were identified as potential OA biomarkers in urine (Table 2). We cannot definitively ascribe these perturbed metabolites to inflammatory OA changes in the knee joint (cartilage, synovium and related tissues) since they may also have a systemic source. Pathway analysis of these metabolites showed that some metabolic pathways were significantly affected in OA participants (adjusted *p*-value < 0.05) including pyruvate metabolism, TCA cycle and amino acid metabolism (Fig. 4). These pathways have been shown to change in previously reported studies of OA using invasive types of samples such as plasma, synovial fluids, serum and cultured synovial tissue (Carlson et al. 2019; Showiheen et al. 2019; Zhai et al. 2018). Our findings indicate that urine, as a readily accessible biofluid, can provide a reliable and comparable metabolic signature of OA and may aid in the understanding of OA pathogenesis and progression of structural change.

### Perturbed pyruvate and TCA cycle pathways in OA

The increased levels of acetylphosphate, fumarate and s-lactoylglutathione in the urine of inflammatory OA participants compared to non-OA controls indicate an enhanced activity of the pyruvate pathway and the TCA cycle possibly due to perturbed metabolism in the cartilage cells. Most of the enzymes involved in the pyruvate metabolism and TCA cycle are located inside the mitochondrial matrix of the cartilage cells. Abnormal urinary excretion of these pathway intermediates provides a metabolic evidence of mitochondrial dysfunction of the cartilage cells in OA as previously reported (Blanco et al. 2004; Gavriilidis et al. 2013). Li and co-workers, linked the detection of abnormal levels of aconitic acid and citric acid in the urine of OA patients to the enhanced activity of the TCA cycle (Li et al. 2010). However, these metabolites were detected in OA patients with no significant difference, which may be attributed to the fact that they used GC–MS for the analysis, and hence, different sensitivity. The increased activity in the pyruvate metabolism was consistent with the level of pyruvate found in the urine of OA patients as it was highly consumed and therefore, lower levels were detected in the urine of OA patients.

### Oxidative stress and amino acid metabolism in OA

Oxidative stress and inflammation processes are believed to play an important primary role in the development and progression of OA (De Ceuninck et al. 2011). Amino acids, the structural building blocks of proteins, play an important role in the regulation of these processes. For instance, L-cysteine is essential for the production of the antioxidant glutathione, which is thought to help in scavenging the destructive oxygen-free radicals produced during normal cell metabolism and plays an important role in the inflammatory response in OA (Surapaneni & Venkataramana, 2007). Under metabolic stress, L-cysteine is produced from homocysteine and 2-hydroxybutyrate is released as a by-product. No significant difference was observed in L-cysteine levels between inflammatory OA participants and non-OA controls, but significantly lower levels of 4-hydroxybutyrate, 3-oxoalanine and homocysteine sulfinic acid were found in the urine of OA participants, which may indicate impaired production or transformation of the necessary L-cysteine in OA. Similarly, abnormal concentrations of urinary amino acids or their metabolites may provide evidence of oxidative stress and/or inflammation in people with OA. Low levels of tryptophan, pipecolic acid, hypoxanthine, aminoadipic acid (lysine metabolites), L-homoserine (serine metabolite) and 3-methylcrotonylglycine (glycine metabolite) were found in the urine of inflammatory OA participants compared to non-OA controls, signalling the possibility of altered metabolic pathways and the biological functions of these amino acids in OA. Oxidative stress is present in several diseases and we cannot be sure that these changed pathways are specific to knee OA as they could be due to systemic effects of other comorbidities.

The increased level of 3-nitrotyrosine in the urine of the inflammatory OA participants further support the oxidative stress in OA. 3-Nitrotyrosine was detected in human urine and plasma by GC–MS and LC–MS and it is strongly believed to be one of the biomarkers of oxidative damage of peroxynitrite (Gaut et al. 2002; Tsikas et al. 2012). Cellular exposure to peroxynitrite was reported to cause calcium dysregulation, mitochondrial dysfunction, inhibition of prostaglandin formation, imbalance of anti-inflammatory mediator pathways and amino acids nitration (Szabo et al. 2007). Therefore, different pathological conditions such as inflammation, pain, arteriosclerosis and neurodegenerative disorders are believed to be associated with peroxynitrite (Pacher et al. 2007). The results obtained here may be an indication of an increased oxidative damage of cartilage cells due to peroxynitrite.

### Perturbation of glutamine metabolism in OA

The eightfold increase in urinary excretion of 2-keto-glutaramic acid, a deaminated metabolite of glutamine, in inflammatory OA participants may be an indication of disturbed glutamine metabolism in the chondrocytes. Normally, excess glutamine conjugates with active phenyl acetate to form N-phenylacetyl-glutamine and Coenzyme A. The end product, N-phenylacetyl-glutamine is then excreted in urine as a normal metabolite of glutamine and phenyl acetate (Shockcor et al. 1996). Alternatively, under abnormal conditions, glutamine is deaminated to form 2-keto-glutaramic acid. The increased levels of 2-keto-glutaramic acid and the decreased level of N-phenylacetyl-glutamine in the urine of inflammatory OA participants, may give further evidence of the altered glutamine metabolic pathways and supported with a previously reported study (Li et al. 2010). This very large change in level of a single metabolite suggests that this may be useful for further investigation as a biomarker of disease progression. However, we do not know if this is a knee-specific change or whether it is derived from a change in the systemic levels of 2-keto-glutaramic acid.

## Conclusion

The significance of this study is that we used a readily accessible non-invasive biofluid, urine, and performed a rigorous validation and identification of the urinary biomarkers of OA. There are some caveats to this study. Firstly, it is a cross-sectional comparative study undertaken at a single time-point. Prospective studies with serial measures in a well characterised cohort of participants are ideally required to confirm these findings and to determine whether the urinary measures predict variation in structural progression of OA. Secondly, the classification of inflammatory OA was based solely on symptoms and clinical signs rather than the use of ultrasound or MRI to provide quantification. Thirdly, this study included people with established knee OA and radiographic change, and study of people at an earlier stage of OA development would be of great interest. Furthermore, a potentially interesting future direction is to compare the male and female participants separately considering the known higher incidence of OA in females. Finally, this study focused exclusively on knee OA and the generalisability of the findings to OA at other joint sites remains to be established.

Overall, we were able to find distinct urinary metabolites associated with inflammatory knee OA but not with non-inflammatory OA participants compared non-OA controls, which may contribute to the understanding of OA pathogenesis and stimulate interest in urinary surrogate biomarkers of OA. However, we are unable to confirm that these observed changes in urinary profile are a direct result of local metabolic changes due to damage to knee cartilage or synovium. Altered activity in TCA cycle, pyruvate and amino acid metabolism, particularly based on the eightfold change in the metabolite 2-keto-glutaramic acid, can provide a basis to understand disease progression based on mitochondrial dysfunction and collagen destruction in the cartilage cells of people with OA, possibly linked to inflammation and oxidative stress.

## Supplementary Information

Below is the link to the electronic supplementary material.

Supplementary Material 1 (PDF 1,503 KB)

Supplementary Material 2 (XLSX 57 KB )
