# Supplementary material for: Metabolic signatures of osteoarthritis in urine using liquid chromatography‐high resolution tandem mass spectrometry
Source: Metabolomics. 2021 Mar 3;17(3):29. doi: 10.1007/s11306-021-01778-3 (PMC7925472; doi:10.1007/s11306-021-01778-3)
Supplement: Supplementary file 1 — Supplementary Material 1 (PDF 1,503 KB) [file 11306_2021_1778_MOESM1_ESM.pdf]

Online Resource 2

Metabolic signatures of osteoarthritis in urine using liquid chromatography-high resolution tandem mass spectrometry

Salah Abdelrazig[a], Catharine A. Ortori[a], Michael Doherty[b,c,d,e], Ana M. Valdes[b,c], Victoria Chapman[b,d,f] and David A. Barrett[a]

[a]Centre for Analytical Bioscience, School of Pharmacy, University of Nottingham, Nottingham, NG7 2RD, UK

[b]Pain Centre Versus Arthritis, University of Nottingham, Medical School, Queen's Medical Centre, Nottingham, NG7 2RD, UK

[c]School of Medicine, University of Nottingham, Nottingham, NG7 2RD, UK

[d]NIHR Nottingham Biomedical Research Centre, University of Nottingham, Nottingham, NG7 2RD, UK

[e]Centre for Sport, Exercise and Osteoarthritis Research Versus Arthritis, University of Nottingham, Nottingham, NG7 2RD, UK

[f]School of Life Sciences, University of Nottingham, Nottingham, NG7 2RD, UK

Corresponding author:

David A. Barrett

Centre for Analytical Bioscience, School of Pharmacy, University of Nottingham, Nottingham, NG7 2RD, UK
Email: David.barrett@nottingham.ac.uk
Tel: +44 (0) 115 951 5062
Fax: +44 (0) 115 951 5102

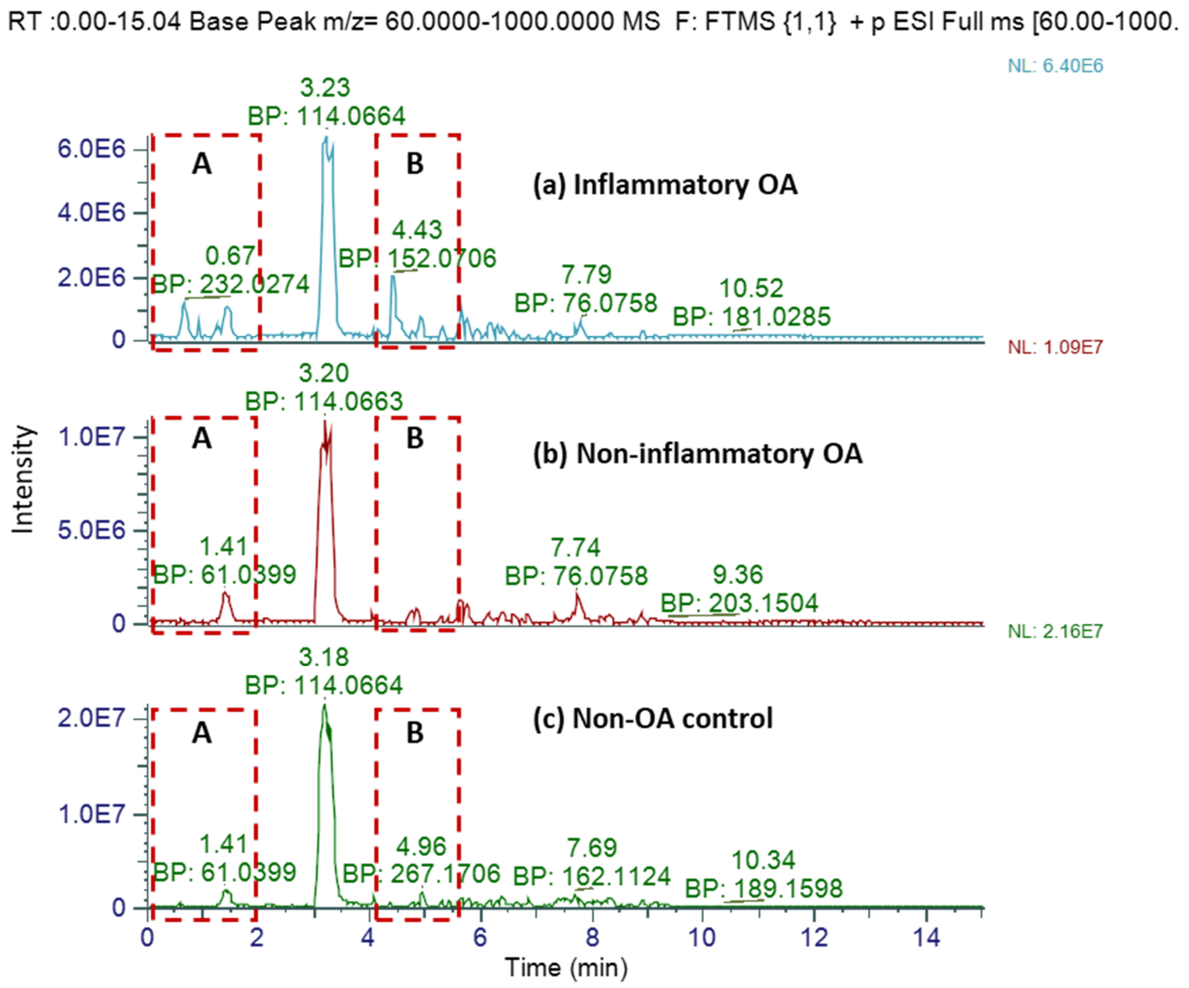

Figure S1 Base peak chromatograms (BPCs) of the urine samples from OA patients and non-OA controls analysed with LC-HRMS. BPCs showed difference between the urine profiles of (a) inflammatory OA participants, (b) non-inflammatory OA participants and (c) non-OA controls in positive ion mode, respectively. The marked regions (A) and (B) show an increased level of the metabolite, *m/z* 232.0274 (unknown) and *m/z* 152.0706 (phenylglycine) in inflammatory OA compared to non-inflammatory OA participants and non-OA controls. The metabolite ion, m/z 181.0286 (ESI+) and, m/z 286.2642 (ESI-) showed higher peak areas in the inflammatory OA compared to non-inflammatory OA participants and non-OA controls. Creatinine, m/z 114.0667 (ESI+) was found to be the most abundant ion in the non-OA controls urine sample.

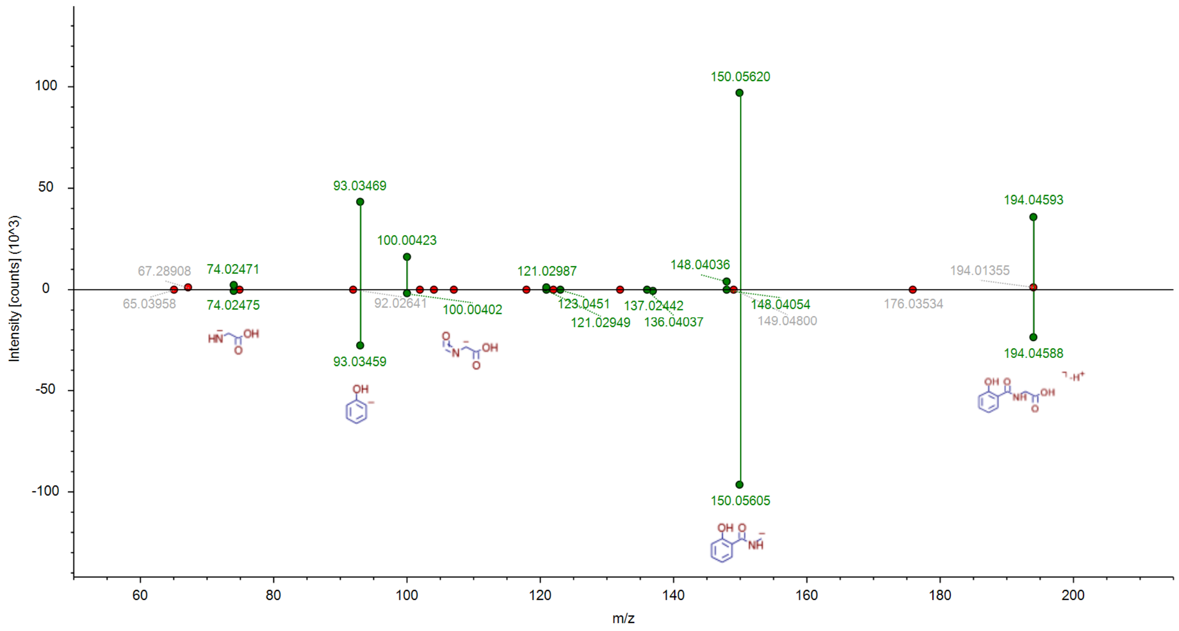

Figure S2 Identification of 2-Hydroxyhippuric acid using *mz*Cloud. Top MS/MS spectrum of the metabolite from the inflammatory OA and non-OA control sample (top) was matched with a reference standard MS/MS spectrum (bottom) from *mz*Cloud database.

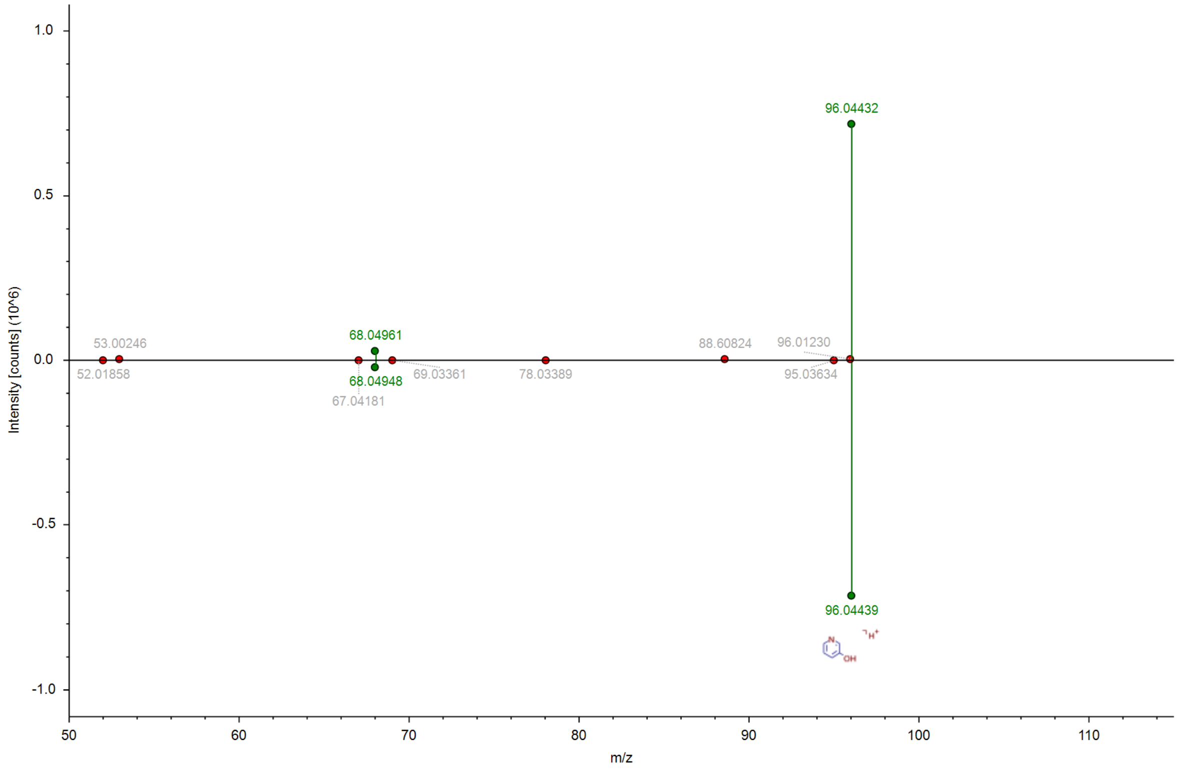

Figure S3 Identification of 3-hydroxypyridine using *mz*Cloud. MS/MS spectrum of the metabolite from the sample (top) was matched with a reference standard MS/MS spectrum (bottom) from *mz*Cloud database.

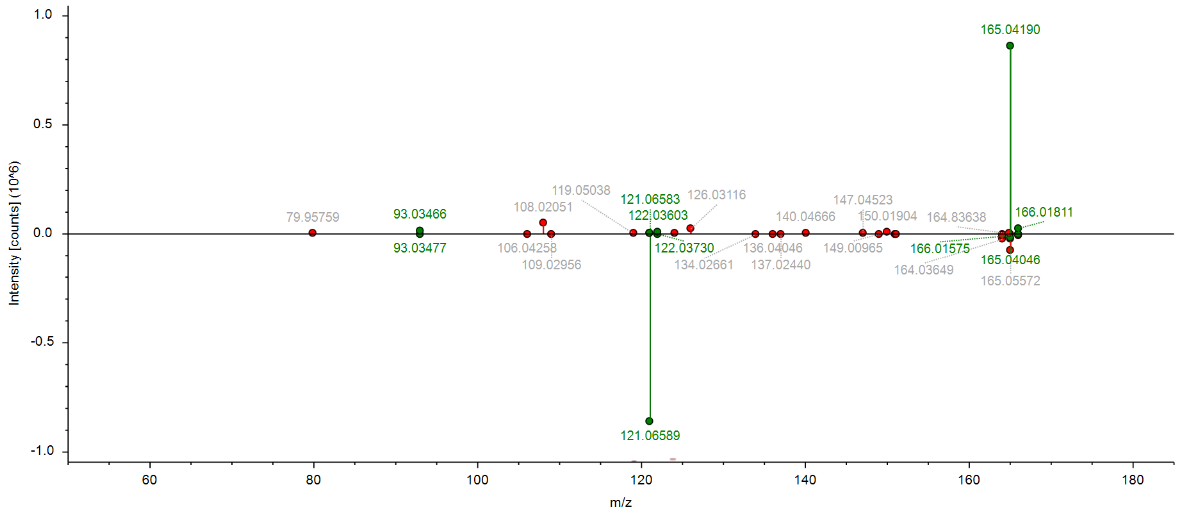

Figure S4 Identification of 3-methoxyphenylacetic acid using *mz*Cloud. MS/MS spectrum of the metabolite from the sample (top) was matched with a reference standard MS/MS spectrum (bottom) from *mz*Cloud database.

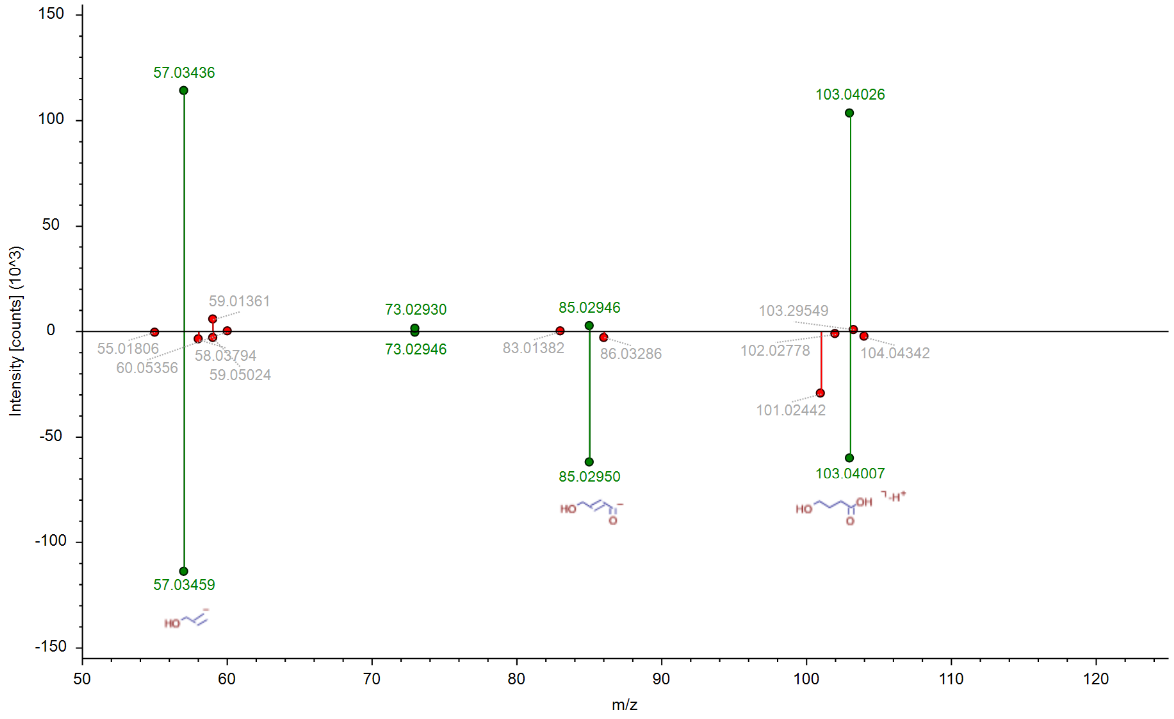

Figure S5 Identification of 4-Hydroxybutyric acid using *mz*Cloud. MS/MS spectrum of the metabolite from the sample (top) was matched with a reference standard MS/MS spectrum (bottom) from *mz*Cloud database.

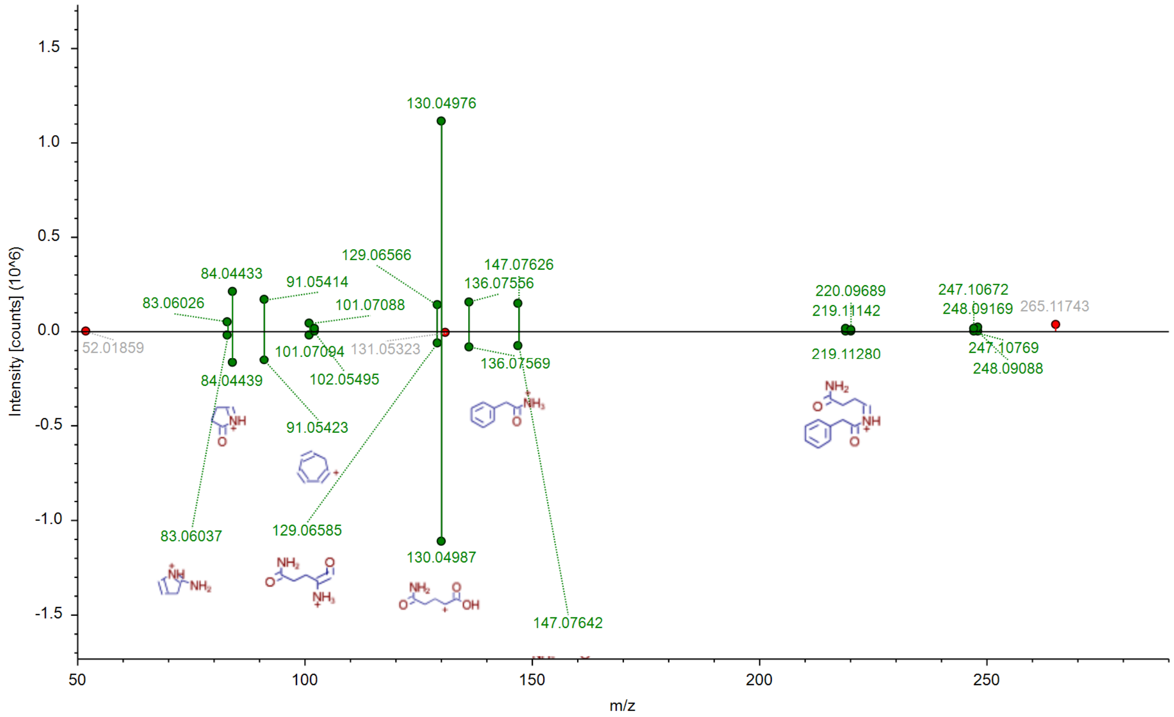

Figure S6 Identification of N-phenylacetylglutamine using *mz*Cloud. MS/MS spectrum of the metabolite from the sample (top) was matched with a reference standard MS/MS spectrum (bottom) from *mz*Cloud database.

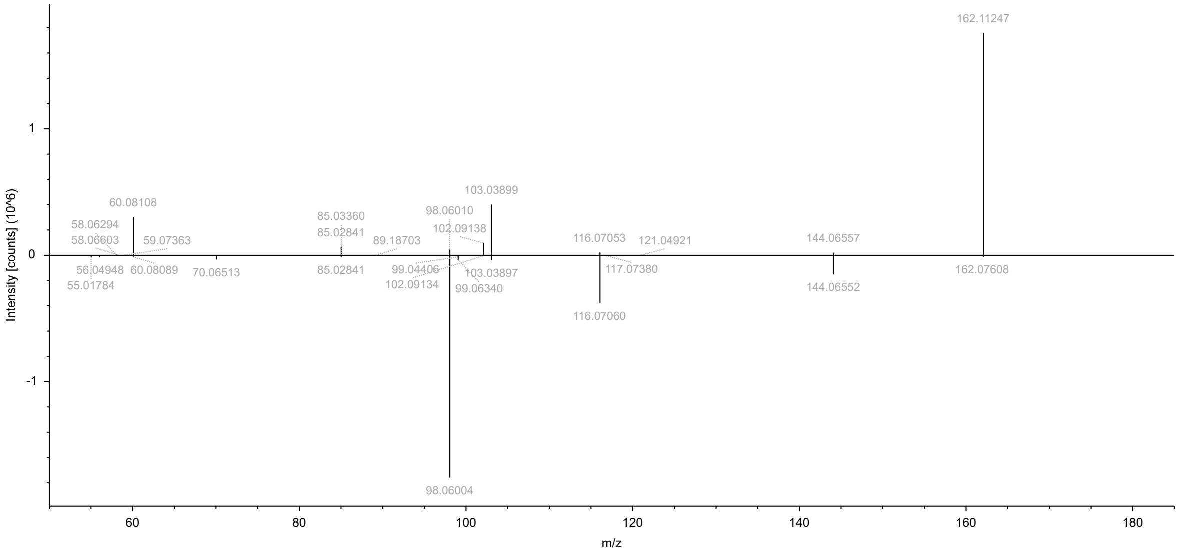

Figure S7 Identification of aminoadipic acid using *mz*Vault. MS/MS spectrum of the metabolite from the sample (top) was matched with a reference standard MS/MS spectrum (bottom) from *mz*Vault database.

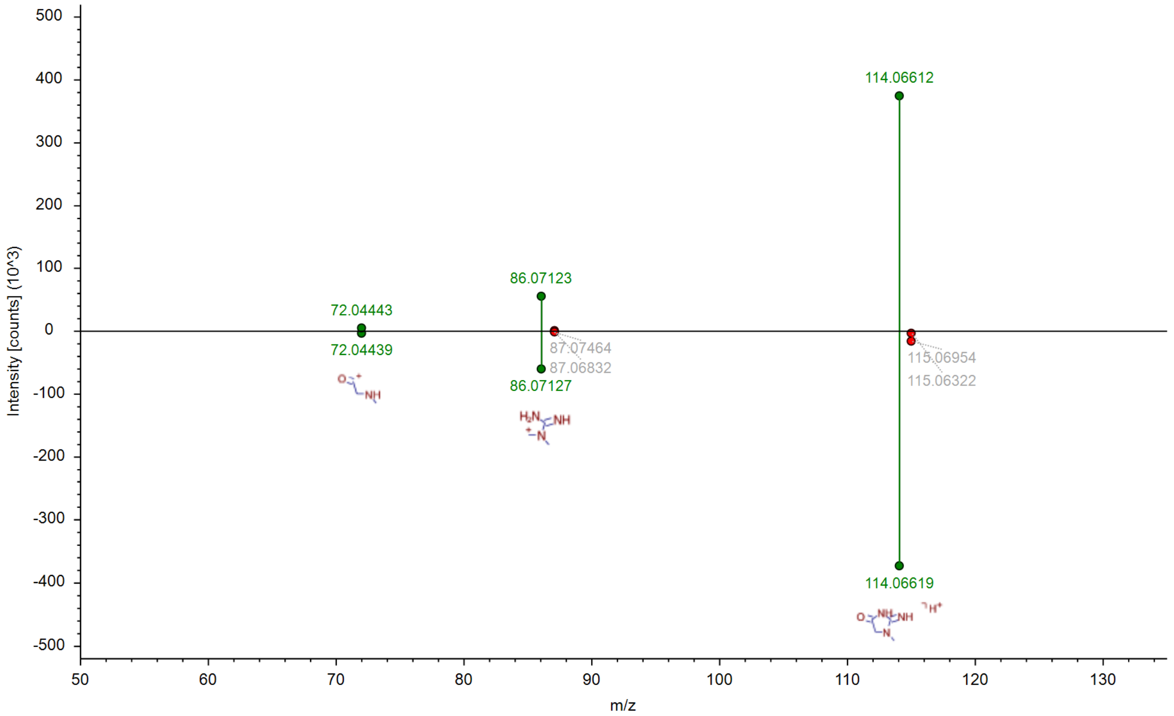

Figure S8 Identification of creatinine using *mz*Cloud. MS/MS spectrum of the metabolite from the sample (top) was matched with a reference standard MS/MS spectrum (bottom) from *mz*Cloud database.

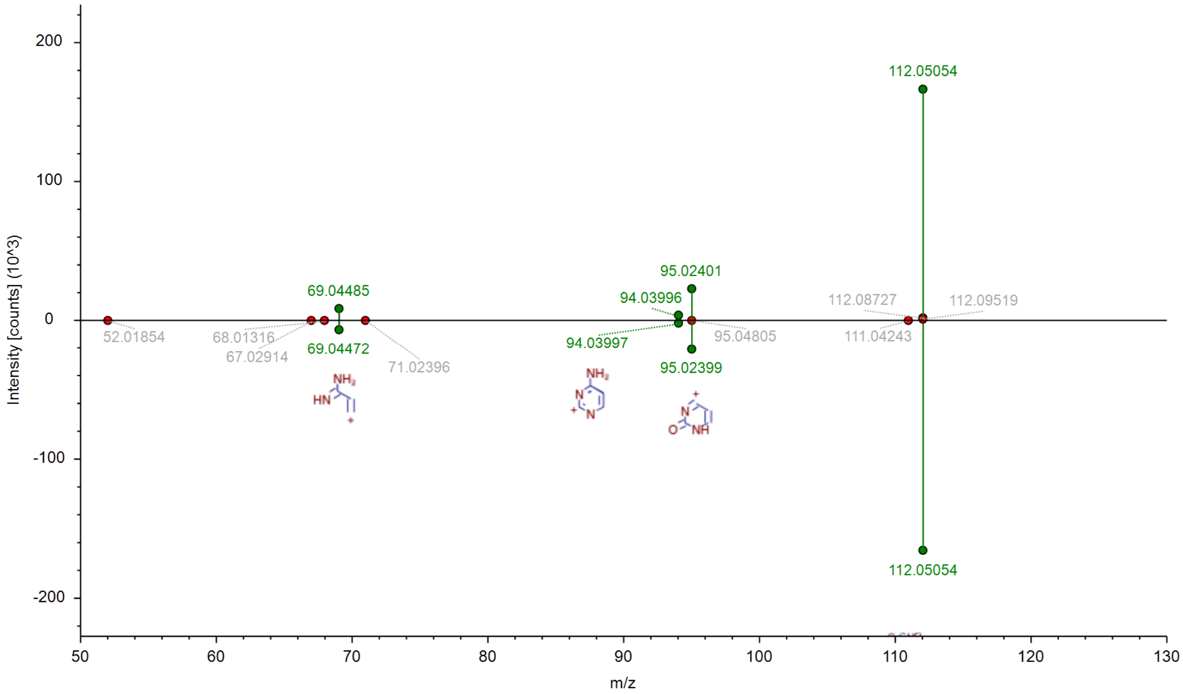

Figure S9 Identification of cytosine using *mz*Cloud. MS/MS spectrum of the metabolite from the sample (top) was matched with a reference standard MS/MS spectrum (bottom) from *mz*Cloud database.

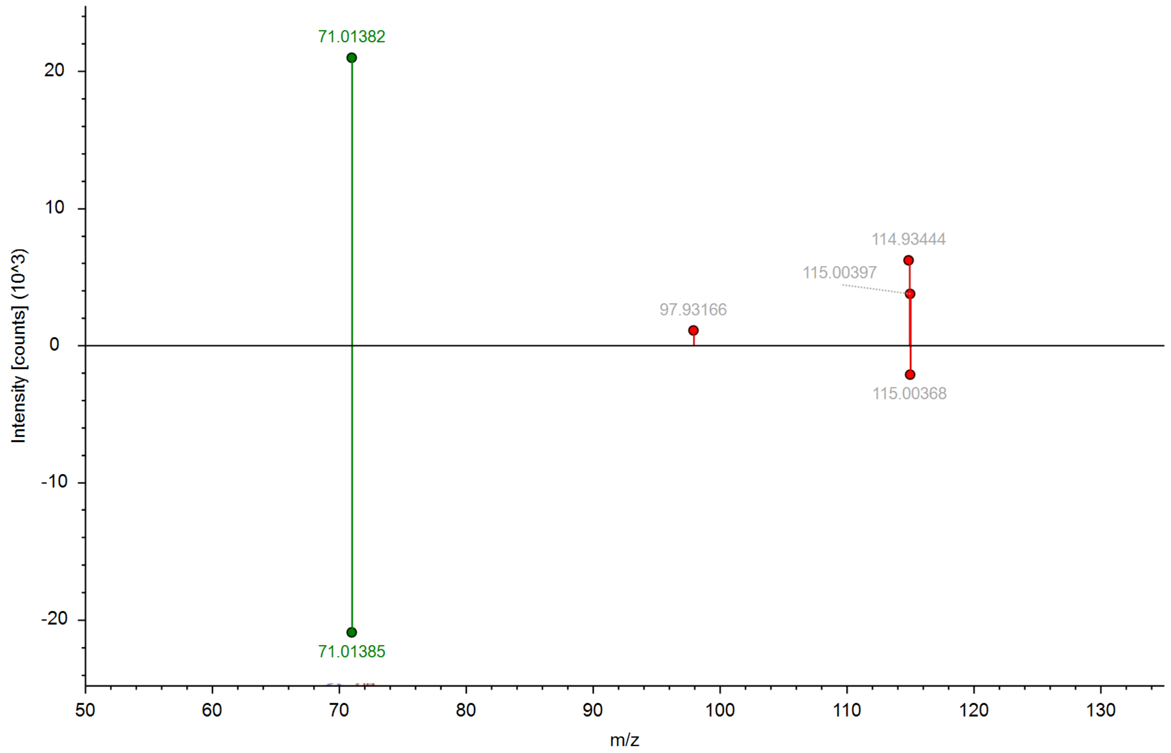

Figure S10 Identification of fumarate using *mz*Cloud. MS/MS spectrum of the metabolite from the sample (top) was matched with a reference standard MS/MS spectrum (bottom) from *mz*Cloud database.

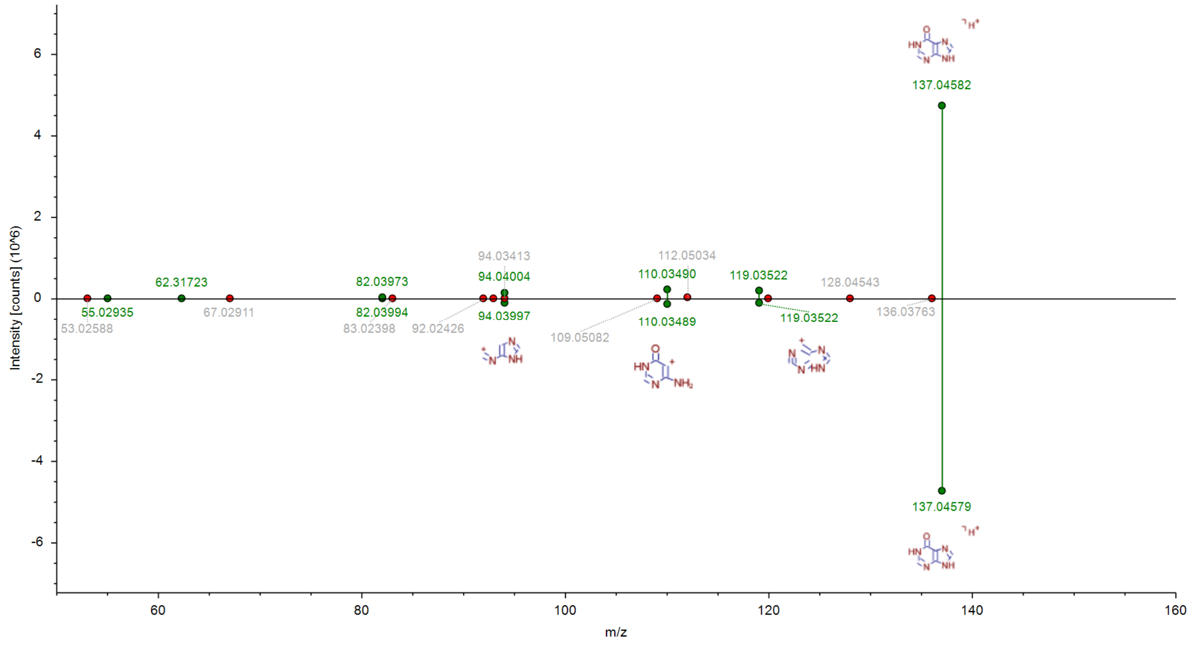

Figure S11 Identification of hypoxanthine using *mz*Cloud. MS/MS spectrum of the metabolite from the sample (top) was matched with a reference standard MS/MS spectrum (bottom) from *mz*Cloud database.

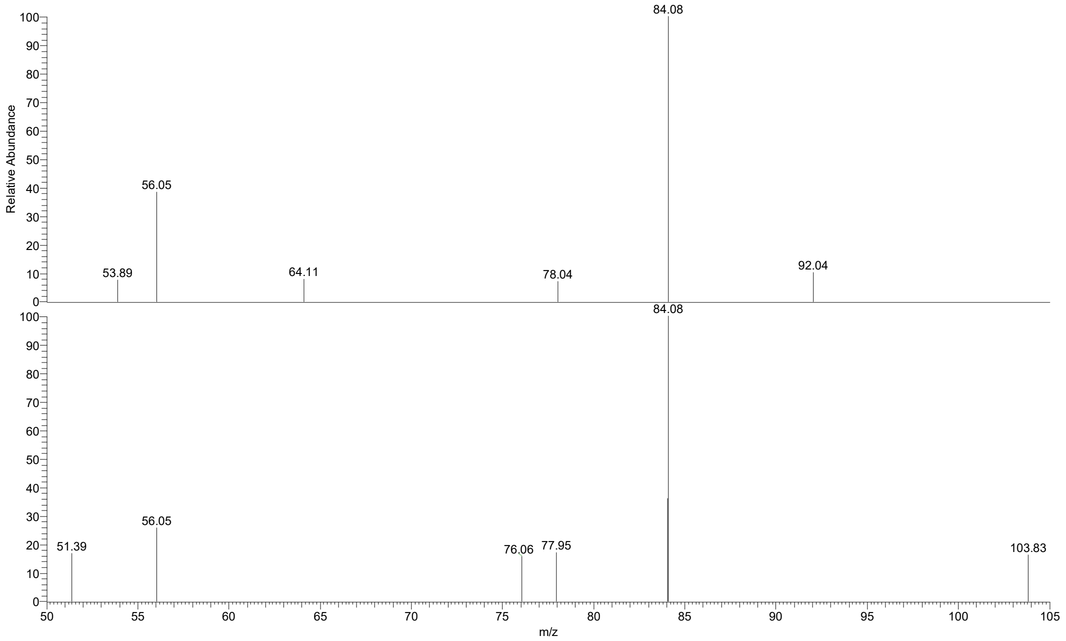

Figure S12 Identification of homoserine using a reference standard. MS/MS spectrum of the metabolite from the sample (top) was matched with a reference standard MS/MS spectrum (bottom).

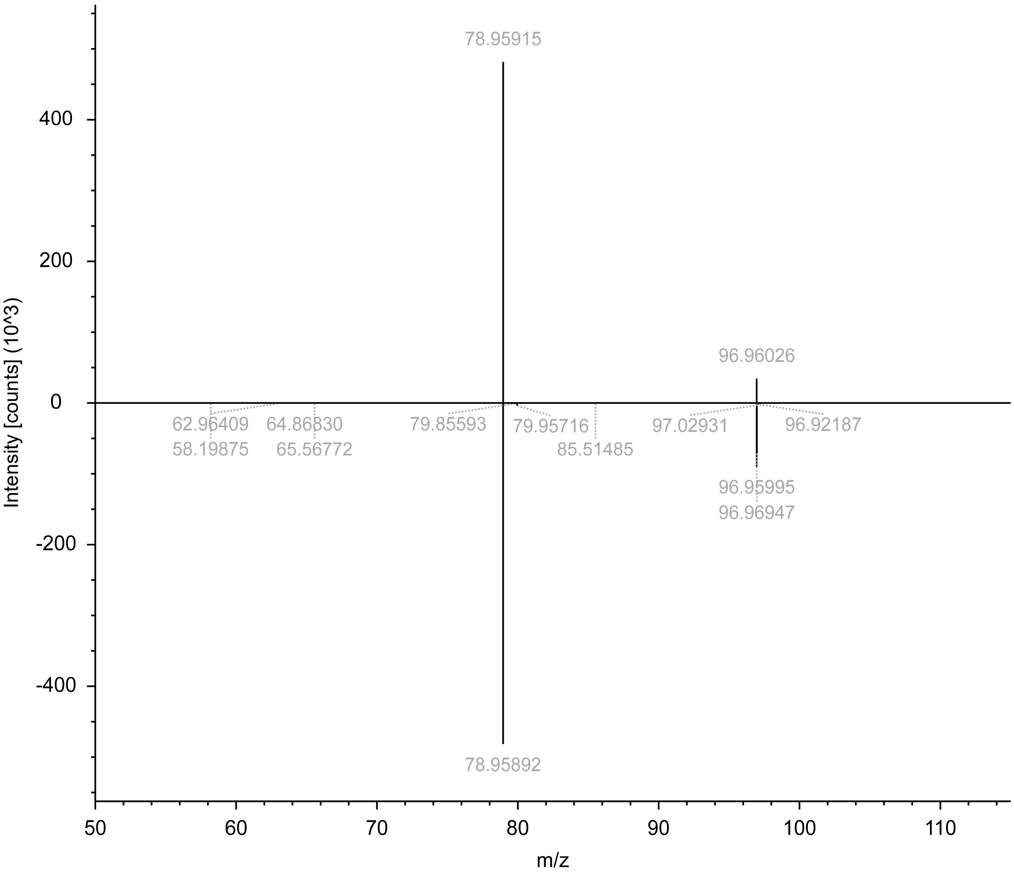

Figure S13 Identification of phosphoric acid using *mz*Vault. MS/MS spectrum of the metabolite from the sample (top) was matched with a reference standard MS/MS spectrum (bottom) from *mz*Vault database.

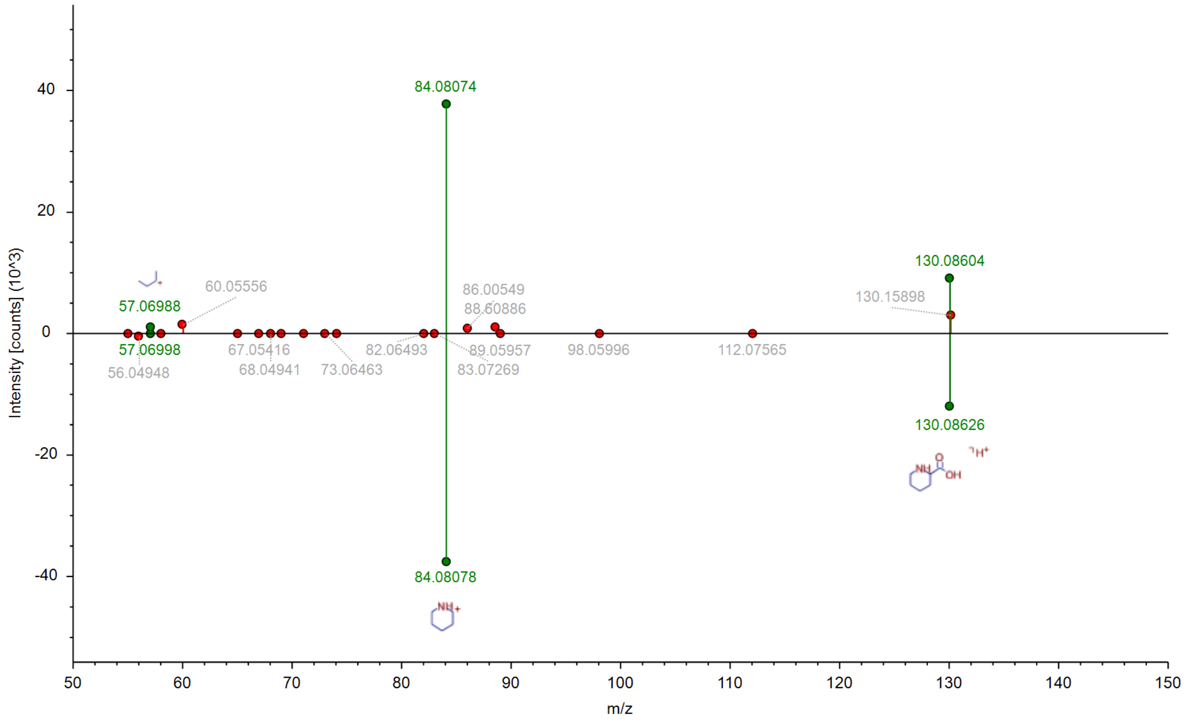

Figure S14 Identification of pipecolic acid using *mz*Cloud. MS/MS spectrum of the metabolite from the sample (top) was matched with a reference standard MS/MS spectrum (bottom) from *mz*Cloud database.

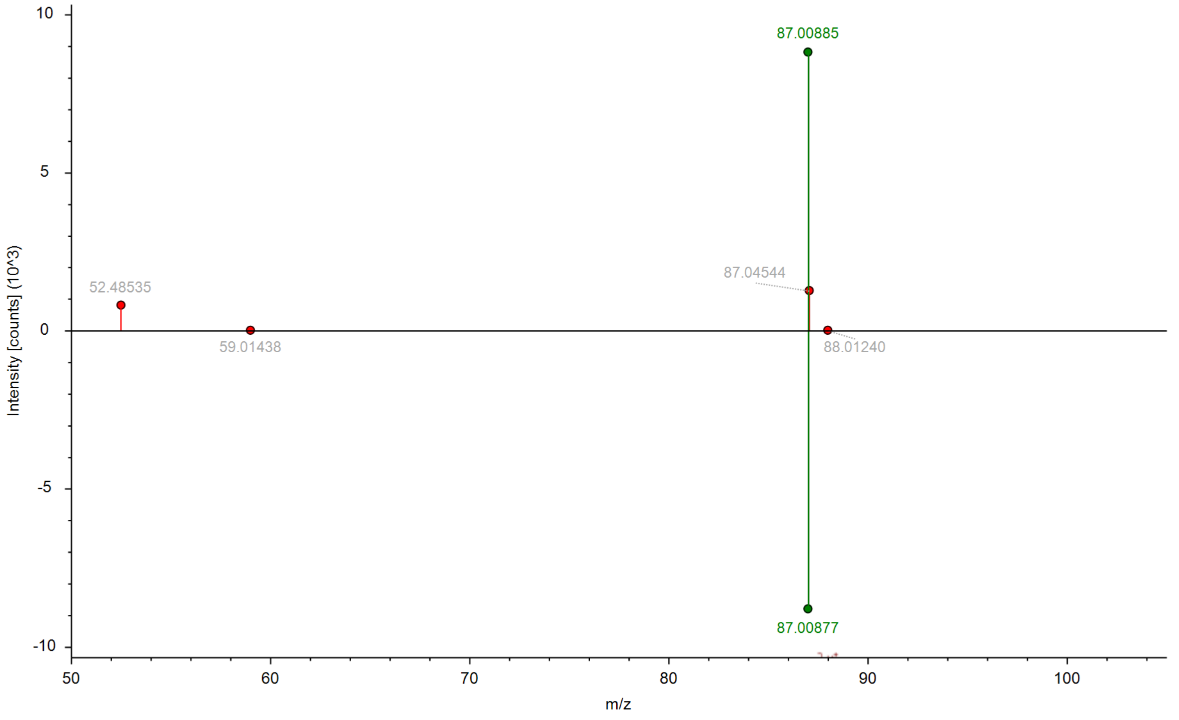

Figure S15 Identification of pyruvic acid using *mz*Cloud. MS/MS spectrum of the metabolite from the sample (top) was matched with a reference standard MS/MS spectrum (bottom) from *mz*Cloud database.

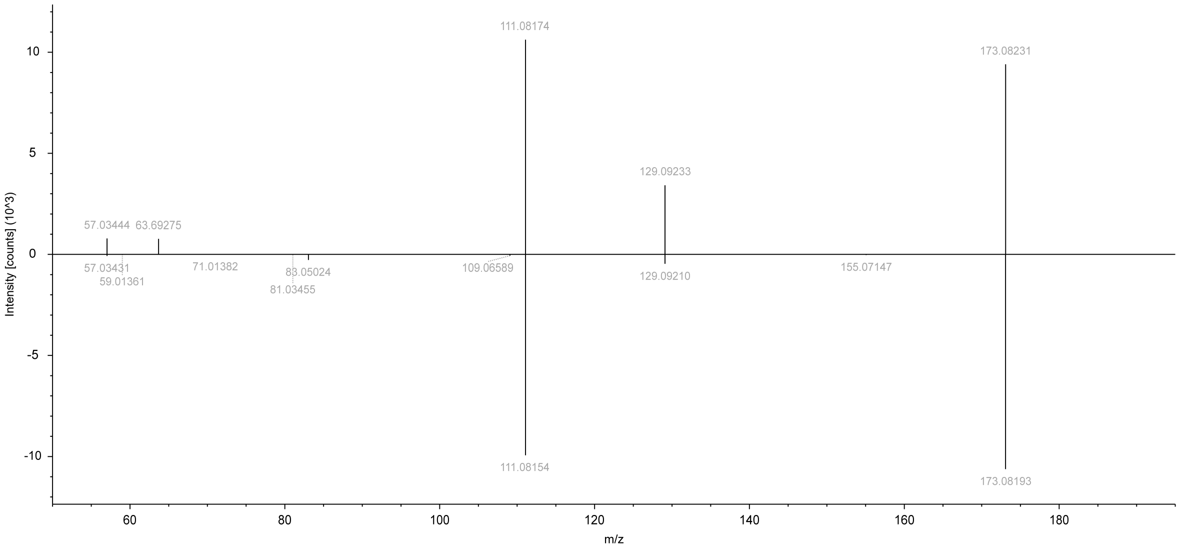

Figure S16 Identification of suberic acid using *mz*Vault. MS/MS spectrum of the metabolite from the sample (top) was matched with a reference standard MS/MS spectrum (bottom) from *mz*Vault database.

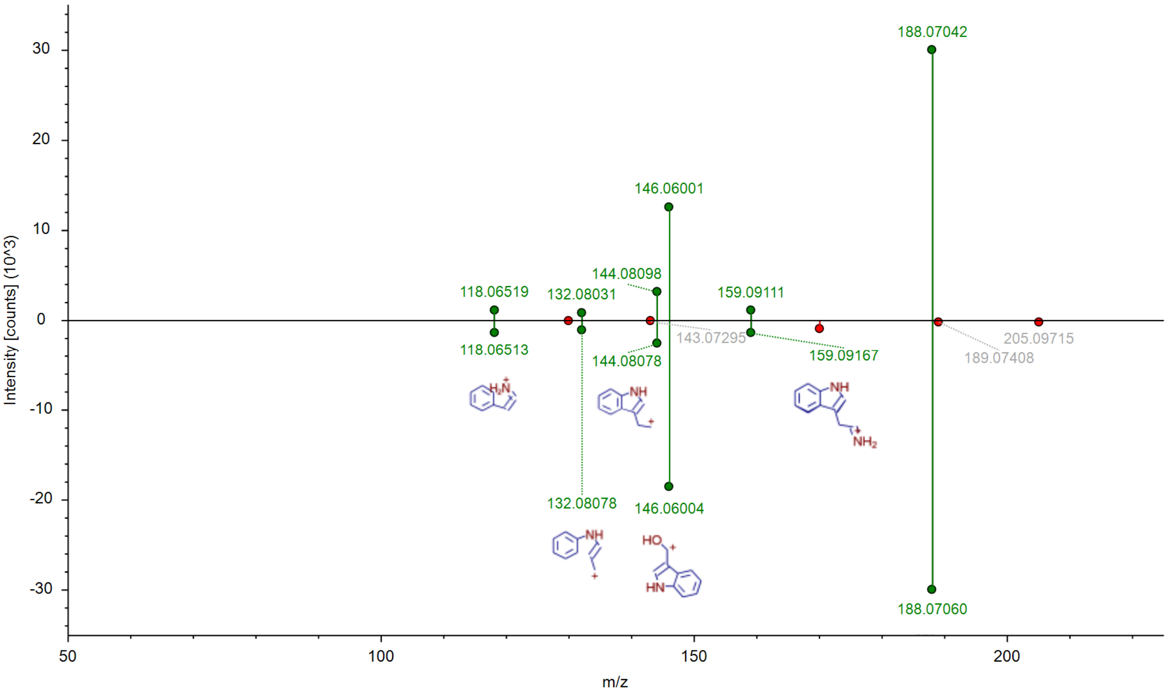

Figure S17 Identification of tryptophan using *mz*Cloud. MS/MS spectrum of the metabolite from the sample (top) was matched with a reference standard MS/MS spectrum (bottom) from *mz*Cloud database.
